# Urinary Trace Element Concentrations in Congolese Patients With Prostate Cancer: A Case‐Control Study

**DOI:** 10.1155/proc/2180096

**Published:** 2026-09-07

**Authors:** Pitchou Mukaz Mbey, Olivier Mukuku, Catherine Ugumba Saleh, Michel Muteya Manika, Pablo Diasiama Kuntima Diangienda, Dieudonné Molamba Moningo

**Affiliations:** ^1^ Department of Surgery, Faculty of Medicine, University of Lubumbashi, Lubumbashi, Democratic Republic of the Congo, unilu.ac.cd; ^2^ Department of Surgery, Urology, Faculty of Medicine, University of Kinshasa, Kinshasa, Democratic Republic of the Congo, unikin.ac.cd; ^3^ Department of Research, Institut Supérieur des Techniques Médicales, Lubumbashi, Democratic Republic of the Congo, istmlubumbashi.net; ^4^ Department of Anaesthesia and Intensive Care, Faculty of Medicine, University of Lubumbashi, Lubumbashi, Democratic Republic of the Congo, unilu.ac.cd

**Keywords:** Democratic Republic of the Congo, prostate cancer, trace elements

## Abstract

**Introduction:**

Prostate cancer (PCa) is one of the most common cancers among men and a major cause of cancer‐related mortality worldwide. In the Democratic Republic of the Congo (DRC), PCa represents an important public health challenge, with limited data available on environmental factors that may be associated with disease occurrence. Recent evidence suggests that trace element exposure may influence prostate carcinogenesis. This study aimed to compare urinary trace element concentrations between PCa patients and age‐matched controls in the DRC.

**Methodology:**

This case‐control analytical study was conducted between April 5, 2018, and April 5, 2023, in Haut‐Katanga Province, DRC. The study included 94 PCa patients and 94 controls matched according to age and sociodemographic characteristics. Urinary concentrations of 12 selected trace elements were measured using inductively coupled plasma optical emission spectrometry. Statistical comparisons were performed using the Wilcoxon–Mann–Whitney U test due to the non‐normal distribution of trace element concentrations.

**Results:**

Compared with controls, PCa patients showed significantly higher urinary concentrations of arsenic, cadmium, cobalt, chromium, copper, manganese, selenium and zinc (*p* < 0.05), whereas aluminium concentrations were significantly lower. No significant differences were observed for nickel, lead or strontium. These findings indicate distinct urinary trace element profiles among PCa patients and controls.

**Conclusion:**

Altered urinary concentrations of several trace elements, including toxic and essential elements, were associated with PCa status in this Congolese population. Because urinary concentrations mainly reflect recent exposure, further studies using complementary biological matrices and longitudinal designs are needed to clarify the biological significance and potential role of trace elements in PCa development.

## 1. Introduction

Prostate cancer (PCa) is one of the most common cancers in men and is a major cause of death worldwide. It is the second most diagnosed male cancer [1, 2], with incidence and mortality varying by region, particularly due to inequalities in access to care [3]. In the Democratic Republic of the Congo (DRC), it is the second most frequently diagnosed cancer after breast cancer, accounting for 14.8% of all newly diagnosed cancer cases. According to the Global Cancer Observatory (GCO/IARC), this cancer represents 12.8% of all cancer‐related deaths, with a 5‐year prevalence of 12.6 per 100,000 population [2]. Limited access to testing and specialised care worsens the prognosis, making this disease a major public health issue.

Despite medical advances, PCa remains a major cause of cancer‐related morbidity and mortality in the DRC [2]. Early detection strategies, although potentially beneficial, remain insufficiently implemented and poorly accessible in routine practice [4]. Consequently, many patients are diagnosed at an advanced stage, limiting therapeutic options and negatively affecting survival outcomes [3]. Hospital‐based studies have reported that 47.1% of patients present with metastatic disease at diagnosis, with a median survival of 30 months and a mean survival of 26.6 months [5]. These findings highlight the urgent need to strengthen PCa screening and management strategies in resource‐limited settings, while identifying factors associated with disease progression and mortality. In this context, Mbey et al. [6] demonstrated that specific trace elements detected in prostate tissue, including strontium, manganese and cobalt, together with clinical characteristics such as age, prostate‐specific antigen (PSA) levels, haemoglobin concentration and metastatic status, were associated with mortality among PCa patients in the DRC.

Among the environmental factors influencing the development of PCa, increasing attention has been directed toward exposure to trace elements. These elements, although present in the body at very low concentrations, may play essential biological roles or become toxic when accumulated beyond physiological levels [7–10]. Several potentially toxic elements, including cadmium (Cd), lead (Pb), nickel (Ni) and arsenic (As), have been implicated in carcinogenesis through mechanisms involving oxidative stress, DNA damage and disruption of cellular signalling pathways [10–12]. In particular, cadmium exposure has been associated with molecular alterations that may contribute to the initiation and progression of PCa [10–12].

Conversely, essential elements such as zinc (Zn) and selenium (Se) may exert protective effects through antioxidant activity, regulation of cellular proliferation and maintenance of genomic stability [7, 13]. However, emerging evidence suggests that the relationship between essential trace elements and PCa risk is more complex than previously assumed. For example, a recent case‐control study among Nigerian men reported a nonlinear association between blood selenium levels and PCa risk, indicating that both insufficient and excessive selenium exposure may influence carcinogenic processes in a context‐dependent manner [14]. Similarly, variations in trace element profiles across populations and environmental settings have been reported. In a Polish population, Drozdz‐Afelt et al. [8] observed significantly higher arsenic concentrations among PCa patients, whereas chromium, zinc, cadmium and lead levels were significantly lower compared with controls, highlighting the heterogeneity of trace element patterns associated with PCa.

Trace elements have been shown to influence not only the development and progression of PCa but also the response to treatment [7]. In particular, cancer cells exhibit an increased demand for copper compared to other tissues, suggesting a potentially exploitable metabolic vulnerability to curb tumour progression [15, 16]. Thus, trace element concentrations play a key role in the balance between cell proliferation and apoptosis. When an imbalance occurs, it can lead to cell damage and promote carcinogenesis [13].

Although most studies investigating the role of trace elements in PCa have been conducted in high‐income countries, recent evidence from African populations suggests that metal exposure may also contribute to PCa risk in sub‐Saharan Africa. In Nigeria, Bede‐Ojimadu et al. reported an association between cadmium exposure and PCa risk, with zinc status modifying this relationship [11]. Similarly, recent case‐control studies from Tanzania have demonstrated associations between urinary heavy metal concentrations, including individual and mixed metal exposures, and PCa risk [17]. Most studies investigating the influence of trace elements in PCa have been conducted in high‐income countries, whereas evidence from Africa remains limited. Recent studies from Nigeria [11, 14] and Tanzania [17] have highlighted the potential role of metal exposure in PCa risk; however, data remain scarce in other African settings, particularly in mining‐intensive regions such as the DRC, where extensive mining and metallurgical activities may contribute to environmental exposure to trace elements. However, exposure to heavy trace elements may differ due to local environmental and dietary specificities. Conducting a comparative analysis between PCa patients and control individuals would help identify possible differences in urinary trace element concentrations and provide new insights into their role in prostate carcinogenesis.

The main objective of this study is to evaluate the differences in urinary trace element concentrations between PCa patients and control individuals. This approach seeks to further our understanding of the role of these elements in prostate carcinogenesis and to investigate their potential as urinary biomarkers for the disease.

## 2. Materials and Methods

### 2.1. Study Setting, Type, Time Period and Population

This case‐control analytical study was conducted between April 5, 2018, and April 5, 2023, in the Haut‐Katanga Province, DRC, in collaboration with several health facilities in the city of Lubumbashi, including the University Clinics of Lubumbashi, the Baraka Arc‐en‐Ciel Center, the Med Park Clinic and the C.M.D.C Polyclinic. The study population comprised adult males aged 47–88 years who were being monitored for PCa, as well as healthy volunteers aged 50–84 years who were matched to PCa patients to maintain similarity in age and other sociodemographic factors. The median age was 69.5 years (IQR: 64.0–75.0 years) among PCa patients and 68.7 years (IQR: 64.0–74.5 years) among controls. The case group was made up of 94 patients who had been diagnosed with PCa, which was confirmed through histopathological examination (prostate biopsy). Diagnosis confirmation was conducted according to standard criteria, which involved elevated PSA levels, clinical symptoms and histopathological evidence. Additionally, the Gleason score was evaluated.

The inclusion criteria for PCa patients were as follows: (i) a confirmed diagnosis of PCa, validated by histological analysis, and (ii) willingness and ability to provide a 24‐h urine sample for trace element analysis. For the 24‐h urine collection, participants were instructed to collect all urine passed over a full 24‐h period, starting after the first morning void and including all subsequent urine up to and including the first void of the following morning. The collected urine was stored in a provided clean, acid‐washed container, kept refrigerated throughout the collection period and subsequently delivered to the laboratory for analysis of trace elements.

Patients had to be free of a recent history of blood transfusion or supplementation with trace elements. The control group consisted of 94 healthy Congolese male volunteers recruited from the general population of Haut‐Katanga Province and not from healthcare facilities. Controls were matched with PCa patients based on age, residence, smoking status, alcohol consumption and family history of cancer. This matching aimed to minimise confounding variables affecting urinary trace element concentrations. Before inclusion, all volunteers completed a health and exposure questionnaire to assess eligibility. All participants had resided in Haut‐Katanga Province for more than 10 years, ensuring comparable environmental exposure conditions related to the mining‐intensive context of the region. Participants in the control group were eligible if they had no history of cancer (including PCa), a normal digital rectal examination of the prostate, a PSA level less than or equal to 4 ng/mL, no previous major urological surgery (such as prostatectomy or genital procedures), no current or recent use of medications or supplements affecting the concentrations of trace elements in the urine, and no known renal or metabolic disorders. Volunteers with significant occupational or environmental exposure to toxic elements were excluded to avoid environmental bias. This thorough selection process ensured the validity of comparisons between the PCa patient and control groups. In addition, a 24‐h urine sample was taken for the determination of trace elements. Urine was selected as the biological matrix because it provides a noninvasive approach for assessing recent trace element exposure and is more feasible for case‐control studies compared with tissue sampling. Although blood and prostate tissue may provide complementary information regarding systemic distribution and local biological effects, urine collection allowed standardised sampling from both PCa patients and healthy controls. PCa patients were diagnosed and followed according to the screening and diagnostic algorithm described by Mbey et al. [4]. Controls underwent prostate assessment, including digital rectal examination and PSA measurement, to exclude undiagnosed PCa and ensure comparability between groups. Comprehensive sampling made it possible to recruit all patients diagnosed and followed for PCa in the targeted health facilities. All participants provided informed consent after being informed of the objectives of the study and the protocols involved. Thus, the participants were rigorously selected to ensure optimal comparability between the two study groups and to minimise biases that could affect the interpretation of the results.

### 2.2. Methods

The biological samples consisted of 24‐h urine samples stored at −20°C at the laboratory of the University Clinics of Lubumbashi in vials containing EDTA for the determination of trace elements. These samples were collected by trained medical personnel and were maintained under these conditions until analysis.

The assays of trace elements were conducted immediately after the thawing of the samples at the provincial laboratory of the Congolese Control Office (OCC) in Lubumbashi, which has been ISO 9150 certified since 2010. Urinary trace element concentrations were measured using the Inductively Coupled Plasma Optical Emission Spectrometry (ICP‐OES) method [Optima 8300 ICP‐OES spectrometer (PerkinElmer, Waltham, USA)].

Briefly, urine samples underwent acid digestion with ultrapure nitric acid before ICP‐OES analysis to ensure adequate mineralisation and quantification of trace elements. Calibration of the ICP‐OES system was performed using a certified multielement calibration standard: TruQms Multi‐Element Calibration Standard 3 (10 μg/mL in 5% HNO_3_), supplied by PerkinElmer (catalogue number: N9301720). Analytical accuracy and quality control were verified using the TruQms Quality Control Standard 21. Quality control procedures included calibration verification and comparison of measured concentrations with certified reference values. Analytical accuracy was confirmed by deviations within acceptable limits (±5%), and spike‐recovery tests demonstrated satisfactory recovery (90%–110%). The quantified elements included aluminium (Al), arsenic (As), cadmium (Cd), cobalt (Co), chromium (Cr), copper (Cu), magnesium (Mg), manganese (Mn), nickel (Ni), lead (Pb), selenium (Se) and zinc (Zn). Detailed validation parameters for the ICP‐OES method, including detection limits, calibration performance, precision, accuracy and other analytical performance indicators, are provided in the Supporting file (available here).

The results of trace element concentrations were expressed as μg/g creatinine. Internal quality controls and calibration procedures were applied throughout the analytical process to ensure the reliability and reproducibility of the measurements.

### 2.3. Statistical Analyses

A database was established in Microsoft Excel, then exported and analysed using Stata version 16. Descriptive analysis of urinary concentrations of trace elements was performed using mean ± standard deviation (SD), medians and interquartile ranges (IQRs). Because the Shapiro–Wilk normality test indicated significant deviations from normality, nonparametric methods were used for group comparisons. The comparison of urinary concentrations of trace elements between PCa patients and controls was conducted using the Wilcoxon–Mann–Whitney *U* test, a nonparametric method suitable for skewed data distributions. To assess associations between urinary trace element concentrations and PCa severity, participants were stratified by PSA levels and Gleason scores. PSA was dichotomised using a 20‐ng/mL threshold, distinguishing localised (≤ 20 ng/mL) from more advanced disease (> 20 ng/mL). Gleason scores were categorised as < 7 (well‐differentiated, less aggressive tumours) or ≥ 7 (poorly differentiated, more aggressive tumours). For each element analysed, the rank sum and rank average were calculated for both groups. The statistical correlation among the trace elements was assessed using the Spearman rank correlation. The resulting *p*‐values were utilised to determine the statistical significance of the observed differences, with a significance level of 5% (*p*  <  0.05) adopted for all analyses to ensure the robustness of the conclusions.

### 2.4. Ethical Considerations

Prior to inclusion in the study, all participants received a detailed explanation of the objectives and conduct of the research. Written informed consent was obtained from each patient, ensuring the confidentiality of the information collected and their right to withdraw from the study at any time, without consequence.

The study was approved by the Ethics Committee of the University of Lubumbashi under the approval number UNILU/CEM/015/2020. It was conducted in strict compliance with the ethical principles set out in the Declaration of Helsinki, as well as the national guidelines of the DRC concerning the ethical review of research involving human subjects. Every precaution was taken to ensure the protection, dignity and confidentiality of the participants throughout the study.

## 3. Results

A preliminary assessment using the Shapiro–Wilk test indicated a significant deviation from normality for all urinary trace element concentrations (Al, As, Cd, Co, Cr, Cu, Mn, Ni, Pb, Se, Sr and Zn) in both PCa patients and controls (*p* < 0.01), reflecting skewed distributions. This justified the use of nonparametric methods, specifically the Wilcoxon–Mann–Whitney U test, for group comparisons. Table 1 presents a detailed analysis of the distribution of urinary trace element concentrations in each group through descriptive statistics (mean, SD, first quartile, median and third quartile) and highlights the differences in central tendency and dispersion for these trace elements studied. Several trace elements (e.g., Al, As, Cd, Co, Cu) showed high SDs relative to their mean concentrations, indicating substantial interindividual variability. This likely reflects heterogeneous environmental exposures among participants, particularly in mining‐impacted areas. The Shapiro–Wilk test confirmed a non‐normal distribution of data, justifying the use of nonparametric statistical methods. It is noteworthy that the distributions of urinary concentrations of trace elements differ significantly between patients and the control group.

**TABLE 1:** Analysis of the distribution of urinary trace element concentrations (μg/g creatinine) in the control (*n* = 94) and PCa patients (*n* = 94) groups, with descriptive statistics.

Trace elements	Control group			PCa patient group		
	Mean ± SD	1st–3rd quartiles	Median	Mean ± SD	1st–3rd quartiles	Median
Al	1.70 ± 1.49	0.40–2.70	1.70	1.18 ± 3.72	0.00–0.00	0.00
As	4.51 ± 3.46	1.70–6.80	4.15	9.06 ± 7.33	4.10–13.10	6.65
Cd	0.02 ± 0.04	0.00–0.00	0.00	3.21 ± 16.40	0.00–0.30	0.10
Co	0.87 ± 0.48	0.50–1.30	0.90	1.72 ± 2.66	0.90–1.60	1.20
Cr	0.25 ± 0.61	0.00–0.20	0.00	0.51 ± 0.69	0.00–0.80	0.10
Cu	27.51 ± 48.88	6.40–27.30	14.15	40.54 ± 48.89	10.40–49.00	18.20
Mn	0.01 ± 0.03	0.00–0.00	0.00	0.38 ± 1.36	0.00–0.20	0.00
Ni	0.10 ± 0.23	0.00–0.00	0.00	0.09 ± 0.29	0.00–0.00	0.00
Pb	0.21 ± 0.45	0.00–0.10	0.00	1.52 ± 10.32	0.00–0.50	0.00
Se	17.10 ± 7.06	10.90–22.20	16.70	25.86 ± 17.11	12.70–31.20	22.40
Sr	5.39 ± 3.63	2.90–7.00	4.30	9.14 ± 10.59	1.30–13.20	4.80
Zn	50.25 ± 49.46	18.80–62.20	38.95	124.63 ± 120.18	28.90–187.90	74.10

*Note:* PCa: prostate cancer.

Abbreviation: SD, standard deviation.

The results of the Wilcoxon–Mann–Whitney test for comparing urinary trace element concentrations between PCa patients and the control group indicate significant differences in value distribution (Table 2). The analyses highlighted various statistically significant differences (*p* < 0.05) between patients and controls in the concentrations of specific trace elements. PCa patients exhibited significantly lower levels of aluminium compared to controls. Furthermore, PCa patients showed higher concentrations of arsenic, cadmium, cobalt, chromium, copper, manganese, selenium and zinc.

**TABLE 2:** Comparison of individual trace element concentrations between PCa patients (*n* = 94) and the control group (*n* = 94) using the Wilcoxon–Mann–Whitney test.

Trace elements	PCa patients		Control group		*p*‐value
	Rank sum	Average rank	Rank sum	Average rank	
Al	6183	65.78	11,583	123.22	< 0.0001
As	10,615	112.93	7151	76.07	< 0.0001
Cd	11,195	119.10	6571	69.90	< 0.0001
Co	10,653.5	113.33	7112.5	75.67	< 0.0001
Cr	9987.5	106.25	7778.5	82.75	0.002
Cu	9802.5	104.28	7963.5	84.72	0.014
Mn	10,544.5	112.18	7221.5	76.82	< 0.0001
Ni	8438	89.77	9328	99.23	0.068
Pb	9470.5	100.75	8295.5	88.25	0.056
Se	10,237.5	108.91	7528.5	80.10	0.0003
Sr	8902.5	94.71	8863.5	94.29	0.958
Zn	10,380.5	110.43	7385.5	78.57	0.0001

In contrast, the differences observed for nickel, lead and strontium were not statistically significant (*p* > 0.05), indicating that the urinary concentrations of these elements did not vary significantly between patients and controls. The levels in both groups appear to be comparable, with no significant influence of PCa patients’ group membership when compared to the control group.

**FIGURE 1:**
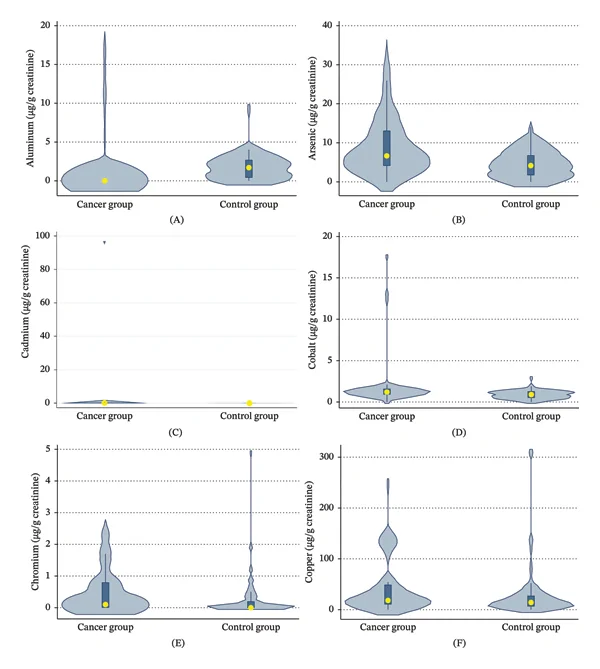
Violin plots of urinary trace element concentrations in prostate cancer patients (study group, *n* = 94) and controls (*n* = 94). (A) Aluminium, (B) arsenic, (C) cadmium, (D) cobalt, (E) chromium, (F) copper. The yellow dot represents the median, the thick line indicates the interquartile range and the whiskers extend to the upper and lower adjacent values. The overlaid curve represents the data density, providing a visualisation of their distribution.

**FIGURE 2:**
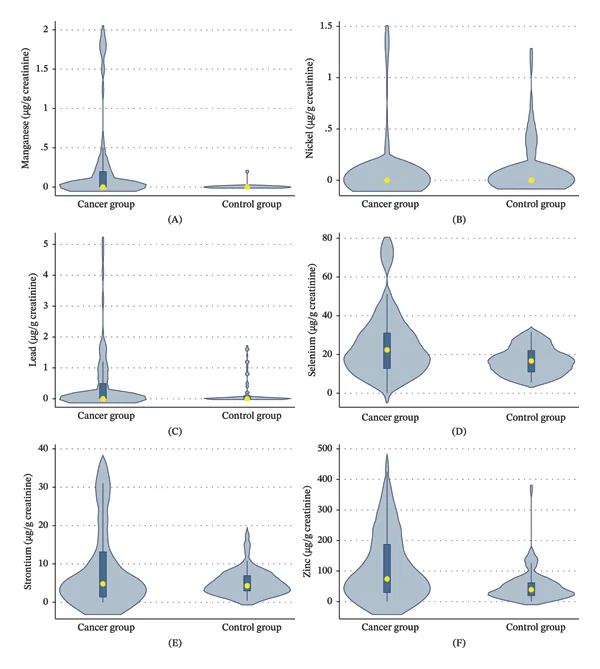
Violin plots of urinary trace element concentrations in prostate cancer patients (study group, *n* = 94) and controls (*n* = 94). (A) Manganese, (B) nickel, (C) lead, (D) selenium, (E) strontium, (F) zinc. The yellow dot represents the median, the thick line indicates the interquartile range and the whiskers extend to the upper and lower adjacent values. The overlaid curve represents the data density, providing a visualisation of their distribution.

Figures 1 and 2 show violin plots of urinary trace element concentrations in PCa patients (*n* = 94) and controls (*n* = 94). The highest urinary concentrations for the PCa patients group compared to the control group were observed for arsenic, as shown in Figure 1B. In contrast, for aluminium, PCa patients have significantly lower urinary concentrations compared to controls, as shown in Figure 1A. Significant differences were also noted for other elements such as cadmium, cobalt, chromium, copper, manganese, selenium and zinc (Figures 1 and 2), where PCa patients had higher urinary concentrations compared to controls, with *p*‐values less than 0.05. However, for nickel and lead (Figure 2B,C), the differences observed are not statistically significant (*p* = 0.068 and *p* = 0.056, respectively). In addition, for strontium (Figure 2E), the rank averages are almost identical between PCa patients and controls (*p* = 0.958).

**FIGURE 3:**
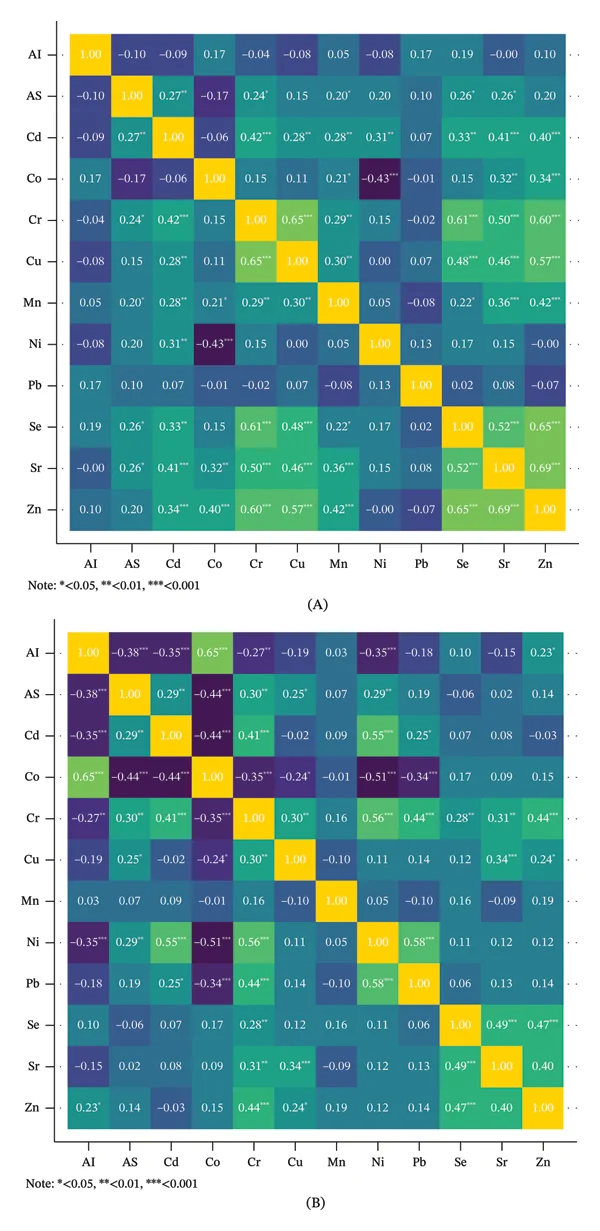
Correlation of urine trace element levels in the prostate cancer group (A) and the control group (B) based on Spearman correlations.

The results of the Spearman correlation analysis between trace elements in the PCa and control groups, as presented in Figure 3, reveal distinct patterns of interdependence for certain elements. These correlations highlight significant differences in the relationships between trace elements within the two groups. In the PCa group, several significant correlations were observed between trace elements, notably chromium with copper (*r* = 0.65, *p* < 0.001), selenium (*r* = 0.61, *p* < 0.001) and zinc (*r* = 0.60, *p* < 0.001). Strontium also showed strong correlations with zinc (*r* = 0.69, *p* < 0.001), chromium (*r* = 0.50, *p* < 0.001), copper (*r* = 0.46, *p* < 0.001) and selenium (*r* = 0.52, *p* < 0.001), highlighting its potential role in a network of interactions among essential elements. Moderate associations were also observed between cadmium and zinc (*r* = 0.34, *p* = 0.001), as well as between cadmium and selenium (*r* = 0.33, *p* = 0.001), indicating shared sources or interactions. Conversely, several weak correlations, such as arsenic and cadmium (*r* = −0.14) and copper and nickel (*r* = −0.05), did not show a significant relationship.

In the control group, several significant correlations were also identified. Cobalt was strongly correlated with aluminium (*r* = 0.65, *p* < 0.001). Nickel was significantly associated with cadmium (*r* = 0.55, *p* < 0.001), cobalt (*r* = −0.51, *p* < 0.001) and chromium (*r* = 0.55, *p* < 0.001), suggesting complex interactions between these elements. Zinc was well correlated with strontium (*r* = 0.40, *p* < 0.001) and selenium (*r* = 0.47, *p* < 0.001). Notable correlations were also observed between lead and nickel (*r* = 0.58, *p* < 0.001). However, weak correlations, such as those between arsenic and cadmium (*r* = 0.30) and between copper and zinc (*r* = 0.01), suggest less interconnection between these elements in the control group.

In PCa patients, the comparison of urinary trace element concentrations between PSA ≤ 20 ng/mL and PSA > 20 ng/mL groups showed no significant differences for most elements (*p* > 0.05), suggesting no association between these trace elements and PSA levels. However, chromium (*p* = 0.0471) and strontium (*p* = 0.0251) demonstrated statistically significant differences (Table 3).

**TABLE 3:** Comparison of urinary trace element levels between prostate‐specific antigen (PSA) level groups using the Wilcoxon rank‐sum test.

Trace elements	PSA ≤ 20 ng/mL (*n* = 18)		PSA > 20 ng/mL (*n* = 76)		*p*‐value
	Rank sum	Average sum	Rank sum	Average sum	
Al	936.5	52.03	3528.5	46.43	0.2859
As	758	42.11	3707	48.78	0.3555
Cd	675	37.50	3790	49.87	0.0719
Co	762	42.33	3703	48.72	0.3749
Cr	656	36.44	3809	50.12	0.0471
Cu	740	41.11	3725	49.01	0.2727
Mn	840.5	46.69	3624.5	47.69	0.8923
Ni	851	47.28	3614	47.55	1.0000
Pb	793.5	44.08	3671.5	48.31	0.5094
Se	760	42.22	3705	48.75	0.3656
Sr	622	34.56	3843	50.53	0.0251
Zn	674.5	37.47	3790.5	49.87	0.0832

In the comparison of urinary trace element levels between Gleason score groups (Table 4), no statistically significant differences were found for any of the elements analysed (all *p* > 0.05), suggesting no clear association between these elements and histological aggressiveness in this cohort.

**TABLE 4:** Comparison of urinary trace element levels between Gleason score groups using the Wilcoxon rank‐sum test.

Trace elements	Gleason score < 7 (*n* = 15)		Gleason score ≥ 7 (*n* = 79)		*p*‐value
	Rank sum	Average sum	Rank sum	Average sum	
Al	677	45.13	3788	47.88	0.5756
As	633.5	42.23	3831.5	48.51	0.4147
Cd	584	38.93	3881	49.13	0.1697
Co	610.5	40.70	3854.5	48.80	0.2913
Cr	613	40.87	3852	48.80	0.2907
Cu	740.5	49.37	3724.5	47.18	0.7725
Mn	636	42.40	3829	48.49	0.3732
Ni	679.5	45.30	3785.5	47.89	0.5416
Pb	692.5	46.17	3772.5	47.74	0.8119
Se	651	43.40	3814	48.30	0.5254
Sr	612.5	40.83	3852.5	48.76	0.3018
Zn	624.5	41.63	3840.5	48.64	0.3636

## 4. Discussion

In this study, we compared urinary concentrations of trace elements in Congolese PCa patients and healthy controls. Significant differences were observed for several elements, including arsenic, cadmium, cobalt, chromium, copper, manganese, selenium and zinc. PCa patients exhibited higher levels of these elements, except for aluminium, which showed lower concentrations compared to healthy controls. These results indicate that specific trace elements may be involved in prostate carcinogenesis, serving either as risk factors or as short‐term exposure markers for the disease.

The results of this study provide new information on the involvement of trace elements in PCa, particularly in a specific environmental context such as that of Lubumbashi, DRC, a region characterised by intensive mining and metallurgical activities that may contribute to human exposure to these trace elements under distinct physicochemical conditions. Few studies from African populations have investigated the relationship between trace element exposure and PCa. Recent case‐control studies from Nigeria and Tanzania have reported associations between urinary or blood concentrations of potentially toxic metals and PCa risk, highlighting the importance of region‐specific environmental exposures in understanding prostate carcinogenesis [11, 14, 17]. The elevated levels of arsenic, cadmium and cobalt observed in PCa patients are consistent with the recognised carcinogenic potential of these trace elements, particularly through mechanisms involving oxidative stress, reactive oxygen species generation, genotoxic damage and disruption of DNA repair pathways, which are well documented in the literature [18, 19]. These alterations may affect cellular homeostasis and molecular processes involved in carcinogenesis, potentially contributing to the malignant transformation and progression of prostate cells.

In our study, the median urinary arsenic concentration was 4.15 μg/g creatinine in healthy subjects and 6.60 μg/g creatinine in PCa patients, suggesting higher arsenic exposure among patients. These concentrations should be interpreted as biomarkers of recent exposure rather than cumulative body burden. Urinary arsenic levels are influenced by factors such as dietary intake, particularly seafood consumption and ideally require arsenic speciation for accurate interpretation. Although the observed concentrations remained below commonly used health‐based reference thresholds, the higher levels in PCa patients suggest differences in exposure patterns that warrant further investigation. Drozdz‐Afelt et al. [8] observed a median plasma arsenic concentration of 0.6 μg/L in their Polish PCa patients, with a lower value of 0.2 μg/L in their Polish control group, which shows a notable difference from our results, probably due to the biological material used, environmental, dietary and ethnic factors specific to each population. Arsenic is a metalloid found in drinking water, soil, plants, fish, cigarettes and cereals, with contaminated water being the main source of poisoning. In Europe, the average daily intake of As ranges from 0.09 to 0.38 μg/kg body weight, with no maximum limit set for food, while the maximum allowable plasma concentration in drinking water is 10 μg/L [20]. In the United States, the average daily arsenic intake is estimated at 12–14 μg, with drinking water containing 10 μg/L representing a major exposure source and the WHO recommended limit [19]. Epidemiological studies have linked arsenic‐contaminated water exposure to increased PCa incidence [21–23]. Supporting these findings, Tyagi et al. reported higher arsenic and cadmium concentrations in prostate tumour tissues than adjacent tissues, suggesting a potential role of metal exposure in PCa‐related molecular alterations, although differences in biological matrices limit direct comparisons with urinary measurements [24]. As promotes the malignant transformation of prostate epithelial stem cells via the suppression of miR‐143, increasing the expression of the antiapoptotic genes B‐cell lymphoma 2 (BCL‐2) and BCL‐XL as well as metalloproteinases (MMP‐2 and MMP‐9) [25]. It activates KRAS (Kirsten Rat Sarcoma Virus Oncogene Homolog) and the RAS/ERK (Rat Sarcoma/Extracellular signal‐Regulated Kinase) pathway, stimulating tumour proliferation [26]. Chronic exposure leads to genomic and mitochondrial instability, promoting oncogenic mutations and oxidative stress. As also impairs the methylation of tumour suppressor genes (RASSF1A and p16) [27] and, in combination with oestrogen, reduces DNA repair. It confers stem properties to cancer cells via the regulation of microRNAs and the inhibition of PTEN (phosphatase and tensin homolog) [19, 28]. Prolonged exposure induces metabolic reprogramming and inflammatory modulation, increasing tumour aggressiveness [29]. Although the duration of exposure could not be specified in our study—since urine reflects only recent exposure and not long‐term accumulation—we chose urine as a biological matrix because it is noninvasive, easy to collect and suitable for monitoring short‐term exposure to trace elements. However, this approach has limitations, as urinary concentrations can vary depending on hydration status and recent intake, and may not fully represent chronic exposure. Despite these constraints, the high concentrations observed in PCa patients highlight the need for further research on prolonged exposure to arsenic, particularly in an environment such as that of Haut‐Katanga province (DRC), in order to assess its role in the development of PCa.

The PCa patients had higher concentrations of cadmium and lead compared to the control group. This observation contrasts with the results of the Polish study by Drozdz‐Afelt et al. [8], who found higher cadmium levels in the control group than in PCa patients. The authors explain this difference by the significantly higher percentage of smokers in the control group of their study, thus highlighting the impact of smoking on concentrations of these elements. The relationship between cadmium and PCa remains controversial. While some meta‐analyses have found no clear association between Cd exposure and PCa risk [9, 10], other studies have linked intratumoural Cd levels to androgen receptor expression, particularly among African‐American men [30]. Evidence from African populations is emerging; studies from Nigeria and Tanzania have reported associations between cadmium or urinary heavy metal exposure and PCa risk, highlighting the influence of environmental and nutritional contexts on metal‐related carcinogenic effects [11, 14, 17]. Mechanistically, Cd may promote prostate carcinogenesis by altering androgen receptor activity, inducing epithelial–mesenchymal transition through TGF‐β (transforming growth factor β) signalling, and promoting cellular transformation and resistance to apoptosis [19, 31]. In mice, Kolluru et al. [31] found that prolonged exposure to Cd promotes the formation of prostate tumours and induces changes in proliferation and invasion markers. Cd, by replacing zinc in prostate tissues, alters the structure of the p53 protein, thus compromising DNA repair and promoting carcinogenesis [19].

The results of our study show that urinary chromium levels were significantly higher in PCa patients compared to control individuals. A meta‐analysis by Krstev and Knutsson [32] indicates that exposure to chromium may increase the risk of PCa. This finding is consistent with previous research indicating that chromium, particularly in hexavalent form (Cr(VI)), plays a role in the progression of PCa. Indeed, several studies have demonstrated that Cr(VI) can promote the proliferation and invasion of cancer cells by activating molecular pathways such as epithelial–mesenchymal transition and androgen receptor signalling via interaction with targets such as Melanoma‐Associated Antigen Family B2 (MAGEB2) [33, 34]. These mechanisms contribute to cell migration and tumour growth. Our findings reinforce the hypothesis that increased exposure to chromium may be linked to a poor prognosis in PCa patients, highlighting the need to further explore the impact of this environmental contaminant on the progression of this disease.

The results of our study show that urinary cobalt levels are significantly higher in PCa patients compared to controls. This finding is consistent with the study by Mbey et al. [6], which also associates high urinary levels of cobalt with an increased risk of mortality. A systematic review suggests that cobalt may play a key role in prostate carcinogenesis and serve as a potential biomarker for this disease [35]. Cobalt could indeed be involved in the biological processes that promote PCa, thus opening up prospects for its use as a diagnostic and prognostic indicator. Additionally, the work of Pietrzak et al. [36] shows that elevated serum cobalt levels are linked to less favourable clinical outcomes, supporting the hypothesis of cobalt involvement in PCa progression. This finding suggests that monitoring cobalt levels could provide valuable prognostic information and guide therapeutic interventions. However, urinary cobalt concentrations primarily reflect recent exposure and may be influenced by occupational, dietary and environmental sources. The absence of established disease‐specific urinary cobalt thresholds limits the interpretation of these findings, and future studies integrating exposure history and occupational assessment are needed.

Our study reveals an increase in zinc and selenium levels in PCa patients. Significant differences were observed between urinary concentrations of these elements in patients and healthy controls, supporting the idea of their role in prostatic carcinogenesis, as suggested by other studies [7, 37]. However, unlike studies based on prostate or plasma tissue, our analysis used urine samples, which could explain some discrepancies. Selenium, which has been studied for its protective effects against PCa, has been associated with a reduced risk in several studies [7, 37]. Nevertheless, recent evidence from Nigeria suggests that the relationship between selenium and PCa may not be linear. Bede‐Ojimadu et al. reported a U‐shaped association between blood selenium concentrations and PCa risk, indicating that both low and excessive selenium levels may influence carcinogenic pathways depending on the biological and environmental context. Our results show elevated urinary selenium levels in PCa patients, but we caution that this may not reflect a direct protective effect on prostate tissue, as urinary selenium concentrations do not capture long‐term exposure or tissue‐specific activity. Zinc, essential in cell regulation and antioxidant defence, has shown similar findings: higher levels have been associated with a reduced risk of PCa, demonstrating the antitumour properties of zinc in PCa [7]. The correlation between zinc and selenium is consistent with their shared role in antioxidant defence. However, using urinary rather than prostate or plasma tissue concentrations may limit the accuracy of these results. More research is needed to better understand the impact of these elements on the pathophysiology of PCa.

As reported in the study by Zhan et al. [37], one of the most surprising findings of our study is the reduction in aluminium levels observed in PCa patients, which contrasts with previous reports suggesting that aluminium may be a contributing factor to various types of cancer. Previous studies have demonstrated an association between aluminium and disruptions in the cancerous microenvironment, particularly in breast cancer, where increased aluminium levels have been correlated with oxidative stress and pro‐inflammatory inflammation [38]. Additionally, a study in rats showed that exposure to aluminium during the peripubertal period impaired prostate development, suggesting that aluminium may have a detrimental effect on prostate tissue [39]. However, the observed decrease in aluminium in patients with PCa in our study requires further investigation to determine whether this phenomenon is specific to this population or whether it reflects broader trends in aluminium exposure and metabolism in PCa.

To our knowledge, this study represents one of the first investigations assessing urinary trace element concentrations among PCa patients and controls in the DRC, providing preliminary evidence from a mining‐intensive African setting where environmental exposure data remain limited. Enhancing environmental regulations and refining the control of industrial emissions and contaminants in water and food are paramount in mitigating health hazards. Implementing regular monitoring programs, especially for susceptible populations like industrial workers and residents in polluted regions, is essential for timely risk detection. The study suggests that trace elements, including chromium, cadmium and arsenic, could provide important information on environmental exposures that may contribute to the development of PCa. These findings highlight the potential value of reducing exposure to these elements as a preventive measure. Interventions such as dietary recommendations or specific treatments aimed at minimising exposure to harmful trace elements could play a role in reducing the risk of PCa. Importantly, urinary concentrations of trace elements should be interpreted as indicators of exposure rather than definitive measures of carcinogenic risk. Differences in hydration status, renal function, dietary intake and exposure timing may influence urinary biomarkers. Moreover, the lack of metal speciation prevents differentiation between chemical forms with distinct toxicological profiles, representing an important consideration for future research.

This study is distinguished by its rigorous methodology, which includes the utilisation of ICP‐OES spectrometry, a state‐of‐the‐art technology for analysing trace elements in urine samples, ensuring precise and dependable results. The homogeneous study population enables better control of selection biases and helps to minimise result variability.

However, there are several limitations to consider. The observational nature of the study precludes establishing direct causal links between heavy metal exposure and PCa. Confounding factors, such as medical history, lifestyle and diet, were not fully taken into account. Additionally, the lack of detailed exposure assessment, including occupational history, limits the identification of specific exposure sources. Although many participants were retired and had heterogeneous employment histories, occupational activities may represent an important exposure pathway in mining areas. Furthermore, total urinary trace element concentrations were measured without metal speciation, preventing the distinction between chemical forms with different toxicological properties, such as inorganic and organic arsenic. Therefore, specific exposure pathways, including dietary, occupational, environmental, or mining‐related sources, could not be identified, limiting the mechanistic interpretation of our findings. While urine trace element assays are reliable, they primarily reflect recent or short‐term exposure rather than long‐term accumulation in the body. Moreover, although prostate tissue concentrations would provide more precise insight into the biological impact of these elements, it was not feasible to obtain prostate tissue samples from healthy controls due to obvious ethical and practical challenges. This limitation may have affected the depth of our analysis and the ability to directly compare prostate tissue levels between groups, underscoring the need for further research using complementary biological matrices.

Future research should focus on the mechanisms of metal accumulation at the cellular and tissue levels, using prostate biopsies. Longitudinal studies could also track changes in trace element concentrations and their impact on cancer progression, paving the way for more targeted prevention and treatment strategies.

## 5. Conclusion

This study highlights significant differences in the urinary profiles of trace elements between PCa patients and controls in the DRC. Patients had higher levels of arsenic, cadmium, cobalt, chromium, copper, manganese, selenium and zinc, while aluminium was reduced. These results suggest a potential involvement of certain elements in prostate carcinogenesis, either as risk factors or as biomarkers. The identification of toxic elements such as cadmium and arsenic reinforces the hypothesis of an environmental influence. On the other hand, the high levels of zinc and selenium, which have potentially protective effects, require further investigation. These data pave the way for the evaluation of trace elements as urinary biomarkers and targeted prevention strategies.

## Author Contributions

Pitchou Mukaz Mbey, Olivier Mukuku and Dieudonné Molamba Moningo conceptualised the study and designed the methodology; Pitchou Mukaz Mbey, Olivier Mukuku and Pablo Diasiama Kuntima Diangienda developed the software; Pitchou Mukaz Mbey, Catherine Ugumba Saleh and Michel Muteya Manika conducted the validation; Olivier Mukuku performed formal analysis; Pitchou Mukaz Mbey, Catherine Ugumba Saleh and Michel Muteya Manika collected the data; Pitchou Mukaz Mbey, Olivier Mukuku, Catherine Ugumba Saleh and Michel Muteya Manika prepared the original draft of the manuscript; Pablo Diasiama Kuntima Diangienda and Dieudonné Molamba Moningo provided supervision and contributed to the review and editing.

## Funding

This research received no external funding.

## Disclosure

All authors reviewed and approved the final version of the manuscript for publication.

## Ethics Statement

Ethical approval for this study was granted by the Medical Ethics Committee of the University of Lubumbashi (approval number UNILU/CEM/015/2020). Before data collection, written informed consent was obtained from all participants. They were provided with detailed information about the study’s objectives, procedures and the voluntary nature of their participation. Participants were also informed of their right to withdraw at any time without facing any consequences.

The research adhered to the ethical principles outlined in the Declaration of Helsinki and followed the guidelines for the Ethical Review of Research Involving Human Subjects in the DRC, thereby ensuring the protection of participants’ dignity and confidentiality. To maintain anonymity, participants were assigned unique codes rather than their names, preventing individual identification. Additionally, all completed data collection tools were securely stored in a locked location, with access restricted to the principal investigator, ensuring the confidentiality of the information.

## Conflicts of Interest

The authors declare no conflicts of interest.

## Supporting Information

Additional supporting information can be found online in the Supporting Information section.

## Supporting information


**Supporting Information** Supporting Methods: Detailed analytical procedure for urinary trace element determination. Supporting Table 1: Validation Parameters for ICP‐OES Method (Trace Element Analysis). Supporting Table 2: Performance data of the analytical method for inorganic elements in urine.

## Data Availability

The datasets used and/or analysed during the current study are available from the corresponding author upon reasonable request.
