# Supplementary material for: Urinary Trace Element Concentrations in Congolese Patients With Prostate Cancer: A Case‐Control Study
Source: Prostate Cancer. 2026 Sep 7;2026:2180096. doi: 10.1155/proc/2180096 (PMC13548781; doi:10.1155/proc/2180096)
Supplement: Supplementary file 1 [file PROC-2026-2180096-s001.pdf]

SUPPLEMENTARY FILE

Supplementary Table 1: Validation Parameters for ICP-OES Method (Trace Element Analysis)

All detection limits are expressed in μg/L (ppb).

Trace element	Detection Limit (ICP-OES, μg/L)	Comments
Al	1	From standard method using axial view
As	2	
Cd	0.1	
Co	0.2	
Cr	0.2	
Cu	0.4	
Mn	0.1	
Ni	0.5	
Pb	1	
Se	3	
Sr	0.05	
Zn	0.2	

Reference: PerkinElmer. *World Leader in Atomic Spectroscopy: AA, ICP-OES, ICP-MS*. Waltham, MA: PerkinElmer Inc.; 2011. Available online : https://pesystems.cz/wp-content/uploads/2013/03/BRO_WorldLeaderAAICPMSICPMS.pdf

Supplementary Table 2: Performance data of the analytical method for inorganic elements in urine.

Trace element	Wavelength (nm)	LOD (µg/l)	LOQ (µg/l)	$\bar{X}$ (µg/l)	S_r (µg/l)	CV_r (%)	S_{Fi} (µg/l)	CV_{Fi} (%)
Al	167,079	1,73	5,75	120	3,40	2,83	11,60	9,67
As	188,979	4,20	14,00	63,31	3,68	5,81	5,52	8,72
Cd	214,441	0,27	0,90	13,51	0,24	1,78	0,77	5,70
Co	228,616	0,30	1,00	19,43	0,49	2,52	0,92	4,73
Cr	205,559	1,50	5,00	16,22	0,51	3,14	1,42	8,75
Cu	324,754	0,60	2,00	634	7,90	1,25	23,64	3,73
Mg	279,078	5,70	19,00	10120	122,50	1,21	240,20	2,37
Mn	257,61	0,12	0,40	51,05	0,91	1,78	2,56	5,01
Ni	221,648	0,60	2,00	26,7	0,99	3,71	1,97	7,38
Pb	220,353	1,50	5,00	18,37	1,50	8,17	2,87	15,62
Se	196,089	3,00	10,00	12,14	1,20	9,88	1,91	15,73
Zn	213,857	1,80	6,00	123,6	2,30	1,86	6,13	4,96

LOD: Limit of Detection of the method

LOQ: Limit of Quantification of the method

$\bar{X}$: Mean concentration level (excluding outliers) used for repeatability and intermediate precision determinations

S_r: Standard deviation of repeatability

CV_r: Coefficient of variation for repeatability

S_{Fi}: Standard deviation of intermediate precision

CV_{Fi}: Coefficient of variation for intermediate precision
